# A small-molecule allele-selective transcriptional inhibitor of the MIF immune susceptibility locus

**DOI:** 10.1016/j.jbc.2024.107443

**Published:** 2024-06-03

**Authors:** Jia Li, Lin Leng, Georgios Pantouris, Ramu Manjula, Marta Piecychna, Laura Abriola, Buqu Hu, Elias Lolis, Michelle E. Armstrong, Seamas C. Donnelly, Richard Bucala

**Affiliations:** 1 Department of Medicine, Yale School of Medicine, New Haven, Connecticut, USA; 2 Department of Pharmacology, Yale School of Medicine, New Haven, Connecticut, USA; 3 Yale Center for Molecular Discovery, Yale School of Medicine, New Haven, Connecticut, USA; 4 Department of Medicine, Trinity College Dublin, Dublin, Ireland

**Keywords:** MIF, macrophage migration inhibitory factor, ICBP90, MIF polymorphism, MIF allele, autoimmunity, inflammation, precision medicine, transcription inhibitor, microsatellite

## Abstract

Functional variants of the gene for the cytokine macrophage migration inhibitory factor (MIF) are defined by a 4-nucleotide promoter microsatellite (−794 CATT_5-8_, rs5844572) and confer risk for autoimmune, infectious, and oncologic diseases. We describe herein the discovery of a prototypic, small molecule inhibitor of *MIF* transcription with selectivity for high microsatellite repeat number and correspondingly high gene expression. Utilizing a high-throughput luminescent proximity screen, we identify 1-carbomethoxy-5-formyl-4,6,8-trihydroxyphenazine (CMFT) to inhibit the functional interaction between the transcription factor ICBP90 (*namely*, UHRF1) and the *MIF* -794 CATT_5-8_ promoter microsatellite. CMFT inhibits *MIF* mRNA expression in a −794 CATT_5-8_ length-dependent manner with an IC_50_ of 470 nM, and preferentially reduces ICBP90-dependent MIF mRNA and protein expression in high-genotypic *versus* low-genotypic *MIF–*expressing macrophages. RNA expression analysis also showed CMFT to downregulate MIF-dependent, inflammatory gene expression with little evidence of off-target metabolic toxicity. These findings provide proof-of-concept for advancing the pharmacogenomic development of precision-based MIF inhibitors for diverse autoimmune and inflammatory conditions.

Macrophage migration inhibitory factor (MIF) is an immunologic and neuroendocrine mediator with important roles in the initiation and the progression of the systemic immune response (1, 2). Functional polymorphisms in the human gene for MIF (*MIF*), which comprise a variant promoter microsatellite (−794 CATT_5-8_, rs5844572), are linked both to the susceptibility and the clinical severity of different autoimmune, infectious, and oncologic diseases (3, 4, 5, 6, 7). The transcription factor ICBP90 (*a.k.a.* UHRF1) activates *MIF* transcription at the −794 CATT_5-8_ promoter microsatellite, with both ICBP90 binding and the mRNA transcriptional response increased with higher microsatellite repetition (8) (Fig. 1*A*). Notably, the high-expression −794 CATT_7_ *MIF* allele occurs in ∼ 20% of the population, and its association with inflammatory damage and increased mortality from certain infections (3, 4, 5, 9, 10, 11), including COVID-19 (6), have positioned MIF as a common disease susceptibility gene and attractive target for personalized medicine.

**Figure 1:**
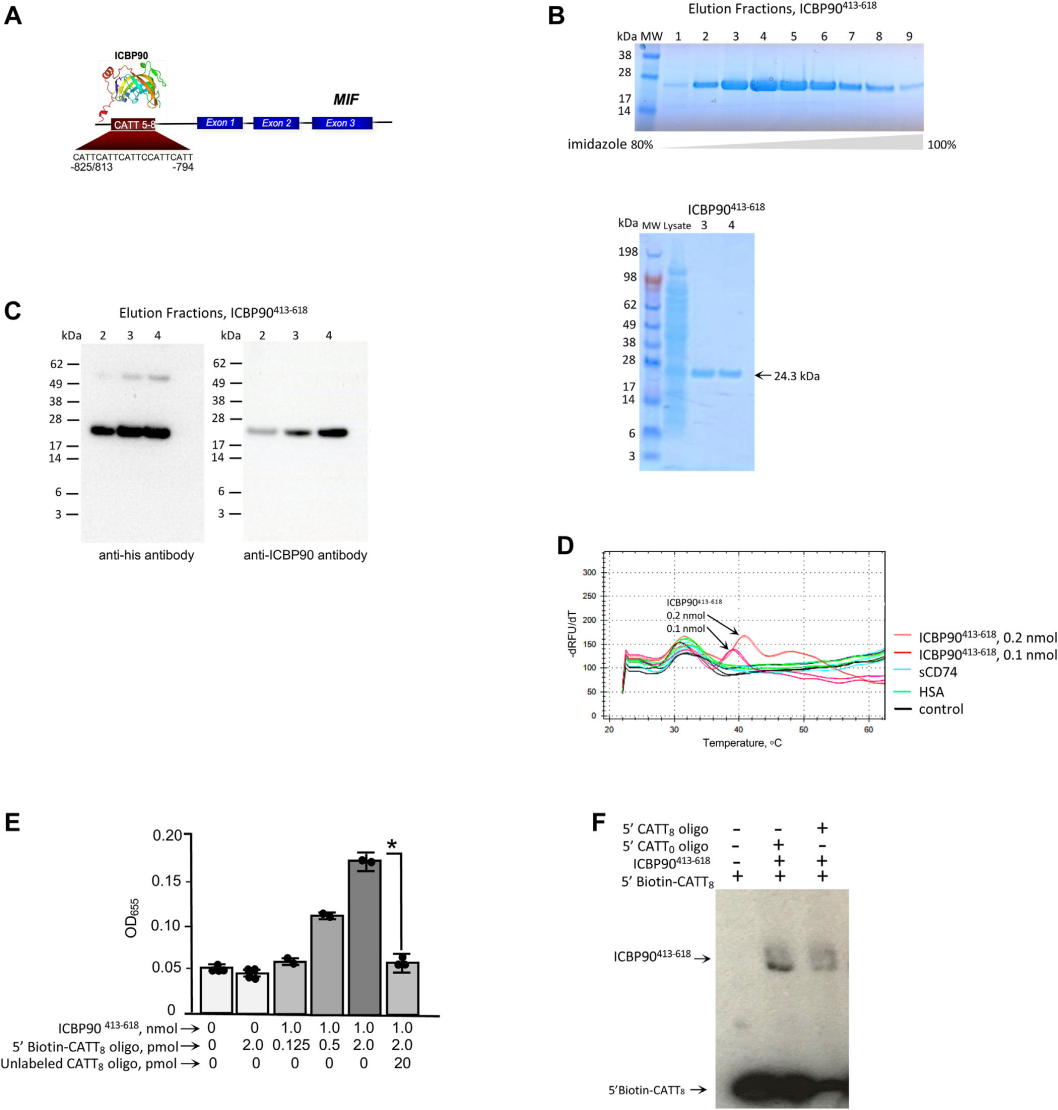
***Development and validation of an interaction assay between MIF promoter microsatellite and ICBP90.**A*, the human *MIF* gene (*rs5844572*) showing its three exons, the −794 CATT_5–8_ promoter microsatellite, and the ICBP90 transcription factor. The numerals refer to nucleotides upstream from the transcription start site. *B*, the *upper panel* shows the gel electrophoretic analysis of FPLC fractions collected by imidazole gradient elution of recombinant histidine-tagged ICBP90^413-618^ (100% imidazole = 500 mM). The *lower panel* shows the purity of the two ICBP90^413-618^ fractions (predicted MW 24.3 kDa) selected for high-throughput screening together with the *E. coli* lysate protein expression starting material. *C*, verification of recombinant ICBP90^413-618^ immunoreactivity by Western blot detection with anti-histidine and anti-ICBP90 antibodies. *D*, melting curve analysis of the −794 CATT_8_ microsatellite DNA (nucleotides −865 to −752) assessed by the fluorescence of the dsDNA binding dye SYBR *green* (λ_max_ 520), showing dose-dependent duplex stabilization by ICBP90^413-618^ (0.2, 0.1 nmol) but not by the control proteins CD74^73-232^ (sCD74) or human serum albumin (HSA) (both at 0.2 nmol). The melting temperature of a corresponding −794 CATT_0_*MIF* promoter DNA (nucleotides −833 to −752) was not affected by ICBP90^413-618^ addition (control). The addition of anti-ICBP90 antibody but not control IgG also prevented melting curve stabilization (*not shown*). *E*, assessment of the stability of the ICBP90 - *MIF* promoter solution interaction by capture ELISA. Recombinant ICBP90^413-618^ was pre-incubated with increasing concentrations of a 5′ biotin-labelled *MIF* promoter CATT_8_ oligonucleotide (0.125–2.0 pmol), with or without excess 20 pmol unlabeled *MIF* promoter CATT_8_ oligonucleotide, followed by addition to immobilized (plate-bound) streptavidin, incubation, washing, and detection with horseradish-peroxidase labeled anti-ICBP90. Values shown are mean ± SD and representative of two independent studies (∗*p* < 0.01 by Student’s *t* test). *F*, electromobility shift assay (EMSA) showing retardation of the electrophoretic migration of a 5′ biotin-*MIF* promoter CATT_8_ oligonucleotide by ICBP90^413-618^ in the presence of a *MIF* promoter oligonucleotide lacking the CATT_8_ microsatellite (5′CATT_0_ oligo) and reduction by the presence of excess *MIF* promoter CATT_8_ oligonucleotide (5′CATT_8_ oligo). All data shown are representative of at least two independent determinations.

The identification of ICBP90 as a *MIF* CATT_5-8_ length-dependent transcriptional activator prompted us to consider the development of *MIF* allele-selective inhibitors, which could be applied in a pharmacogenomic manner to treat conditions that are prone in high-genotypic *MIF*-expressing individuals. We report herein our initial success in the screening and characterization of a prototypic small molecule that interferes with the −794 CATT_5-8_ length-dependent transcriptional activation of the MIF gene.

## Results

### Assay development and validation

We expressed and purified a recombinant ICBP90 DNA binding SET and RING finger-associated (SRA) domain (ICBP90^413-618^) for application to a high-throughput screening assay targeting a *MIF* promoter oligonucleotide containing the −794 CATT_8_ microsatellite (*MIF* gene nucleotides −865 to −752). We subcloned ICBP90^413-618^ into an *E. coli* expression plasmid fused to a C-terminal histidine tag for facile affinity purification by FPLC and isolated the resultant 24.3 kDa protein in a pure and immunoreactive form (Fig. 1, *B* and *C*). We tested for stable solution interaction between his-ICBP90^413-618^ with the *MIF* promoter CATT_8_ oligonucleotide by melting curve analysis using thermal cycling and fluorescence detection of the dsDNA binding dye SYBR green. When compared to irrelevant protein controls, *e.g.*, soluble CD74 (sCD74) and human serum albumin (HSA), the addition of ICBP90^413-618^ increased the melting temperature and solution stability of the double-stranded *MIF* promoter CATT_8_ oligonucleotide (Fig. 1*D*). We also tested for the stability of the ICBP90 - *MIF* promoter interaction by the ability of an anti-ICPB90 antibody to detect such complexes in a transcription factor ELISA, which relies on immobilized streptavidin to capture 5′ biotin-labeled *MIF* promoter CATT_8_ oligonucleotides stably bound to ICBP90^413-618^. Complex pulldown showed dose-dependence with added 5′ biotin-*MIF* promoter CATT_8_ oligonucleotide and competition by excess, unlabeled *MIF* promoter CATT_8_ oligonucleotide (Fig. 1*E*). Finally, we tested for the specificity of recombinant ICBP90^413-618^ binding to the *MIF* promoter CATT_8_ microsatellite by electrophoretic mobility shift assay (EMSA), which is a stringent measure of functional complex formation under electrophoretic conditions. Western blot analysis confirmed the ability of ICBP90^413-618^ to retard the electrophoretic migration of a 5′ biotin-*MIF* promoter CATT_8_ oligonucleotide in the presence of a *MIF* promoter oligonucleotide lacking the CATT_8_ microsatellite (CATT_0_) but not in the presence of excess CATT_8_ containing *MIF* promoter oligonucleotide (Fig. 1*F*).

**Figure 2:**
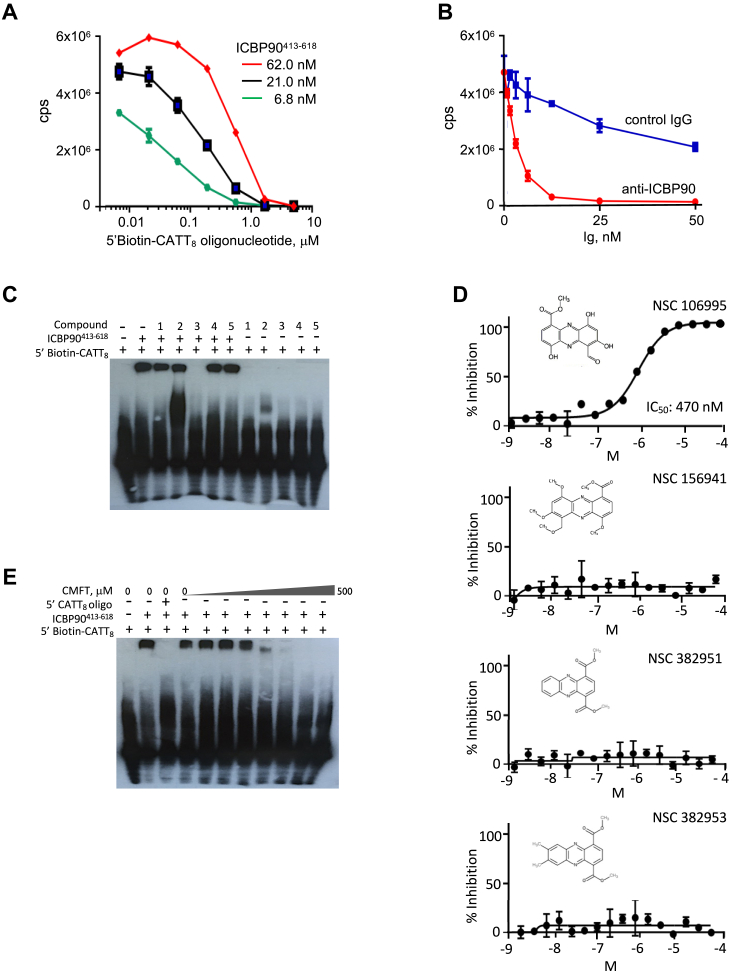
**Development of a high-throughput AlphaScreen for candidate inhibitors of the MIF promoter microsatellite-ICBP90 interaction.***A*, AlphaScreen analyses of the dose-dependent solution interaction of the 5′-biotin *MIF* promoter CATT_8_ oligonucleotide (5′Biotin-CATT_8_, 6.8–5000 nM) with ICBP90^413-618^ at the tested concentrations of 6.8, 21.0, and 62.0 nM. Fluorescence intensity is expressed in counts per second (cps). *B*, dose-dependent inhibition of anti-ICPB90 IgG or an isotypic control IgG antibody on AlphaScreen solution interaction of ICBP90^413-618^ (62 nM) with 5′biotin *MIF* promoter CATT_8_ oligonucleotide (10 nM). *C*, electrophoretic mobility shift assay (EMSA) of five representative compound hits identified by AlphaScreen (Compound 1: NSC 3852, 2: NSC 5159, 3: NSC 106995 (CMFT), 4: NSC 114341, and 5: NSC 11107). The lanes show retention of ICBP90^413-618^/5′Biotin-CATT_8_ molecular complexes, dependence on ICBP90^413-618^, and inhibition by compound 3; Lane 1: 5′Biotin *MIF* promoter CATT_8_ only; Lane 2: 5′Biotin *MIF* promoter CATT_8_ plus ICBP90^413-618^; Lanes 3 to 7: 5′Biotin *MIF* promoter CATT_8_ + ICBP90^413-618^ plus test Compounds 1 to 5; Lanes 8 to 12: 5′Biotin *MIF* promoter CATT_8_ plus test compounds 1 to 5 without ICBP90^413-618^. *D*, dose-dependent inhibition of ICBP90^413-618^/5′Biotin-CATT_8_ solution interaction by CMFT (NSC106995) and 3 structural congeners identified in the screened libraries (NSC 1569419, 382951, 382953). *E*, dose-dependent inhibition by CMFT of ICBP90^413-618^/5′Biotin-CATT_8_ complexation assessed by EMSA. Lane 1: 5′Biotin *MIF* promoter CATT_8_ only; Lane 2: 5′Biotin *MIF* promoter CATT_8_ plus ICBP90^413-618^; Lane 3: 5′Biotin *MIF* promoter CATT_8_ plus ICBP90^413-618^ plus excess 5′ *MIF* promoter CATT_8_ oligonucleotide. Lanes 4 to 11: 5′Biotin *MIF* promoter CATT_8_ plus ICBP90^413-618^ plus increasing concentrations of CMFT (0, 7.8, 15.6, 31.2, 62.5, 125, 250, and 500 μM). Graphed data points are duplicate or triplicate determinations with mean ± SD (Student’s *t* test, two-tailed). EMSA determinations are representative of at least two independent experiments.

For the discovery of candidate inhibitors of ICBP90^413-618^ - *MIF* promoter microsatellite interaction, we applied our test reagents to an amplified luminescent proximity homogeneous assay (AlphaScreen), which detects singlet oxygen (^1^O_2_) emitted from excitation-emission donor-acceptor beads linked to biomolecules of interest (12). This methodology is extremely sensitive, accommodates a broad range of affinities, and is homogenous, which obviates potentially disruptive wash steps. We evaluated assay performance using two buffer conditions and seven concentrations of our two analytes (*e.g., MIF* promoter CATT_8_ oligonucleotide and ICBP90^413-618^ each in the concentration range of 6.8–5000 nM) and selected 10 nM oligonucleotide and 21 nM ICBP90 as optimal (Fig. 2*A*). Using anti-ICPB90 and a control antibody or solvent (0.4% DMSO) as positive and negative controls, respectively (Fig. 2*B*), we performed a pilot screen using the 1600 compound Microsource Pharmakin library. The control well scatter showed stable fluorescent signals across multiple plates with narrow curve width and Z′ scores of 0.84 to 0.93. We selected as an activity cutoff the median signal plus 3 standard deviations (SDs) of compound-containing wells screened at 40 μM; these conditions yielded a workable hit rate of ∼1.7% for the test library.

### High-throughput screening and lead identification

We screened a total of 29,000 compounds from small-molecule collections at Yale’s Center for Molecular Discovery (*e.g.*, ChemBridge, ChemDIV, Enzo, Microsource, NCI, NIH clinical sources). The screened molecules included compounds in the US and International Pharmacopeia, drug-like molecules with known bioactivities and pharmacologically auspicious properties, and diversity and natural product sets. Initial hits were re-screened for assay interference with the AphaScreen TrueHits^Tm^ methodology, which eliminates singlet oxygen and color quenchers, light scatterers, biotin mimetics, and acceptor bead competitors. Compounds with metal chelation properties, for instance, were eliminated for interference with his-ICBP90^413-618^ binding to the Ni^2+^-nitriloacetic (Ni-NTA) acceptor beads. Twenty compounds that scored positively at an initial concentration of 40 μM and survived elimination by TrueHits testing were further screened for dose dependence at 5, 10, 20, and 40 μM concentrations, with solution interaction then verified by competition with excess *MIF* promoter CATT_8_ oligonucleotide and by anti-ICBP90 antibody (tested at 25 nM) (*data not shown*).

A representative EMSA analysis for five of the highest activity compounds revealed 1-carbomethoxy-5-formyl-4,6,8-trihydroxyphenazine (CMFT, NSC#106995) (Fig. 2*C*, *compound 3*), with a solution IC_50_ of 490 nM (Fig. 2*D*), to inhibit ICBP90 interaction with the 5′ containing *MIF* promoter CATT_8_ oligonucleotide in a dose-dependent fashion (Fig. 2*E*). Three structurally related congeners were identified in the screened libraries but showed no activity by solution interaction or by EMSA (Fig. 2*D*, *and data not shown*). Notably, CMFT has been identified in prior high-throughput screening for HIV replication inhibitors and for inhibition of the myotonic dystrophy Muscleblind-like protein interaction with RNA repeats (13, 14), which are observations that support its potential for nuclear uptake.

### Molecular model of CMFT bound to ICBP90

**Figure 3:**
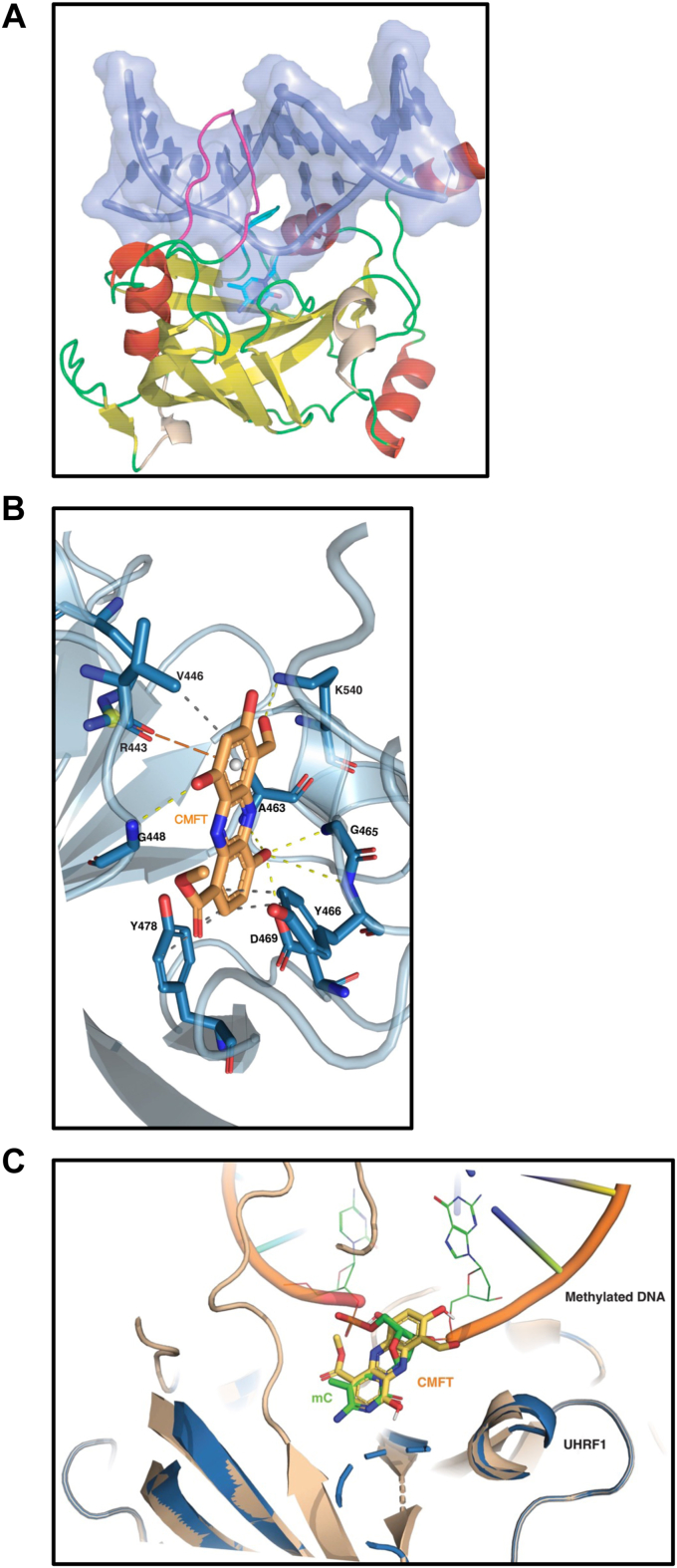
**Structural modeling of CMFT bound to ICBP90 and interaction with DNA.***A*, X-ray co-crystal structure of the ICBP90 SRA domain in its DNA-bound form showing flexible loops (*green*), β-strands (*yellow*), and α-helices (*red*). The duplex DNA is shown as a surface representation with backbone atoms and bases (*blue*) together with the interdigitating NKR (asparagine, lysine, and arginine) motif (*magenta*) (15). *B*, an energy-minimized structure of the SRA domain (*aqua*) docked with CMFT (*orange*), showing hydrogen bonding interactions (*yellow dashes*) with D469, G448, A463, G465, Y466, and K540. The hydrophibic and ionic interactions are shown in *grey* and *orange dashes*, respectively (*C*). Superimposed structures of the SRA domain bound to DNA double helix (*orange*) from the 3CLZ.pdb, modeled CMFT bound SRA (*yellow*) and methylated cytosine (*green*).

To better understand how CMFT interacts with ICBP90 to block DNA binding, we created and refined a structural model using the ICBP90 SRA domain complexed to hemimethylated DNA (3CLZ.pdb) (Fig. 3*A*) (15). The coordinates for DNA were removed, AutoDock was used to identify a site for CMFT within the SRA domain, and the CMFT-SRA complex was energy-minimized. Residues that form interactions with CMFT were analyzed with PLIP (https://plip-tool.biotec.tu-dresden.de/plip-web/plip/index) (16). There are six hydrogen bonds between CMFT and the ICBP90 SRA domain (Fig. 3*B*). Among these interactions are four de-lοcalized hydrogen bonds from the hydroxyl group of the methyl 4-hydroxybenzoate moiety with a backbone amide of Ala-463, two backbone atoms from Gly-465, and a carboxylate oxygen from Asp-469. On the opposite side of CMFT is a 2,4-dihydroxylbenzaldehyde moiety that makes several interactions to stabilize the position of the CMFT at the location where the 5-methylcytosine is flipped out of the duplex DNA from the SRA-DNA complex (Fig. 3*C*). These include a π-cation interaction with Arg-433 and hydrogen bonds with the backbone nitrogen of Gly-448 and the charged side chain nitrogen of Lys-540 with the hydroxyl group and aldehyde group, respectively (Fig. 3*B*). For comparison, 5-methylcytosine also makes five hydrogen bonds with both side chain oxygen atoms of Asp-469 as well as the carbonyl of Thr-479, and backbone amides of Ala-463 and Gly-464. Neither Tyr-466 nor 479 make a π-π interaction with the 5-methylcytosine, and there are no arginine or lysine to make a π-cation interaction. The comparison based on the model of CMFT and the structure of 5-methylcytosine bound to the SRA domain suggests the CMFT makes a stronger interaction than the flipped methylated deoxycytidine.

### Functional inhibition of MIF transcription

**Figure 4:**
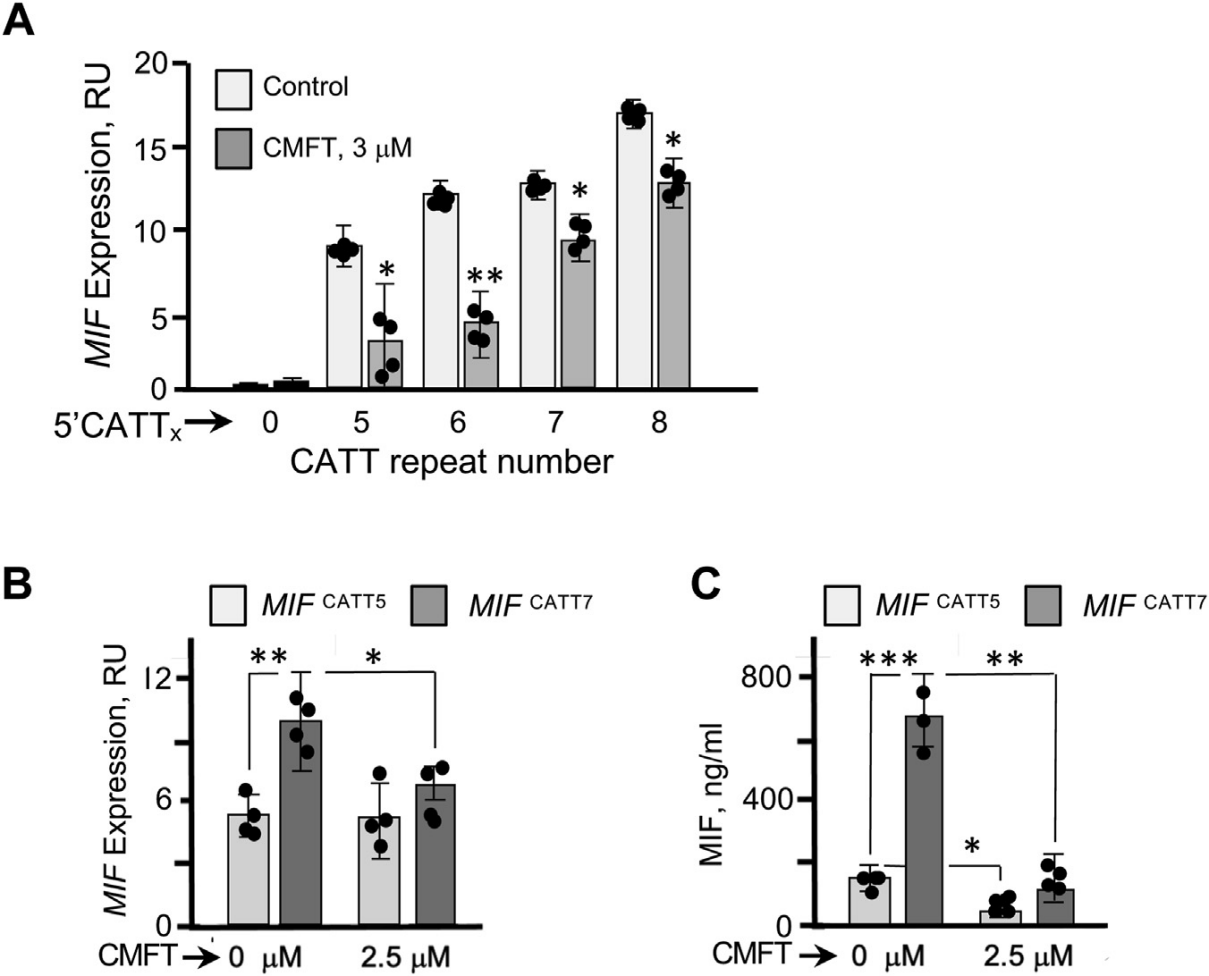
**Impact of CMFT on cellular MIF RNA and protein expression.***A*, human THP-1 monocytes transfected with *MIF* promoter-luciferase reporter plasmids bearing 0, 5, 6, 7, and 8 CATT repeats were stimulated with lipopolysaccharide (LPS, 100 ng/ml), treated with CMFT (3 μM) or vehicle control (0.4% DMSO) for 6 h and luciferase activity assessed by Dual-Luciferase assay. *B*, quantitative PCR analysis of *MIF* mRNA of bone marrow-derived macrophages (BMDMs) isolated from humanized *MIF*^CATT5^ and *MIF*^CATT7^ mice, stimulated *in vitro* with LPS (100 ng/ml), and treated with CMFT (2.5 μM, 6 h) or vehicle control (0.4% DMSO). *C*, ELISA analysis of human MIF in supernatants (triplicate measurements) of cultured LPS-stimulated (100 ng/ml) BMDMs from *MIF*^CATT5^ and *MIF*^CATT7^ mice 6 h after treatment with CMFT (2.5 μM) or vehicle control (0.4% DMSO). Data are the mean+SD and representative of two replicated experiments (∗*p* < 0.05, ∗∗*p* < 0.01, ∗∗∗*p* < 0.001 by Student’s *t* test, two-tailed).

We first assessed the biologic activity of CMFT in cell-based studies using cultured human THP-1 monocytes transfected with variant *MIF* promoter-luciferase reporter plasmids, a methodology used previously to quantify −794 CATT_0-8_ length-dependent mRNA transcription in response to inflammatory stimuli. Following initial dose-ranging studies, the addition of CMFT at 3 μM was observed to reduce 5′CATT_0-8_ length-dependent *MIF* promoter activation in stimulated THP-1 monocytes (Fig. 4*A*).

We recently described a “humanized” *MIF* mouse created by the recombinant replacement of mouse *Mif* with the human low (−794 CATT_5_) and high (−794 CATT_7_) expression *MIF* alleles, thus producing *MIF* ^CATT5^ and *MIF* ^CATT7^ mice, respectively. The utility of a humanized *MIF* gene mouse model is supported by the high sequence conservation of the human and mouse MIF proteins, which share 90% amino acid sequence identity, their interchangeability in mouse and human cell-based assays (17, 18), and by the high sequence conservation between ICBP90 and its mouse homolog Np95 (95% identity in the DNA binding domain) (8). We prepared bone marrow-derived macrophages from *MIF* ^CATT5^ and *MIF* ^CATT7^ mice and stimulated them with gram-negative bacterial lipopolysaccharide (LPS) to induce *MIF* mRNA expression. As expected from prior work (8), inflammatory activation stimulated *MIF* mRNA and MIF protein expression with increased expression observed in macrophages derived from the *MIF* ^CATT7^ *versus* the *MIF* ^CATT5^ mice. Notably, the addition of CMFT to this cell-based assay reduced both *MIF* mRNA and MIF protein expression (Fig. 4, *A* and *B*). There was a 30% reduction in stimulated *MIF* mRNA expression in high-genotypic *MIF*^CATT7^ *versus* low-genotypic *MIF*^CATT5^ BMDMs, which showed no detectable reduction in *MIF* expression upon CMFT treatment (Fig. 4*B*). The impact of CMFT was more evident at the level of MIF protein production, as measured by ELISA of cultured BMDM supernatants, with CMFT reducing MIF protein by >75% in high-genotypic *MIF* ^CATT7^ cells (Fig. 4*C*).

**Figure 5:**
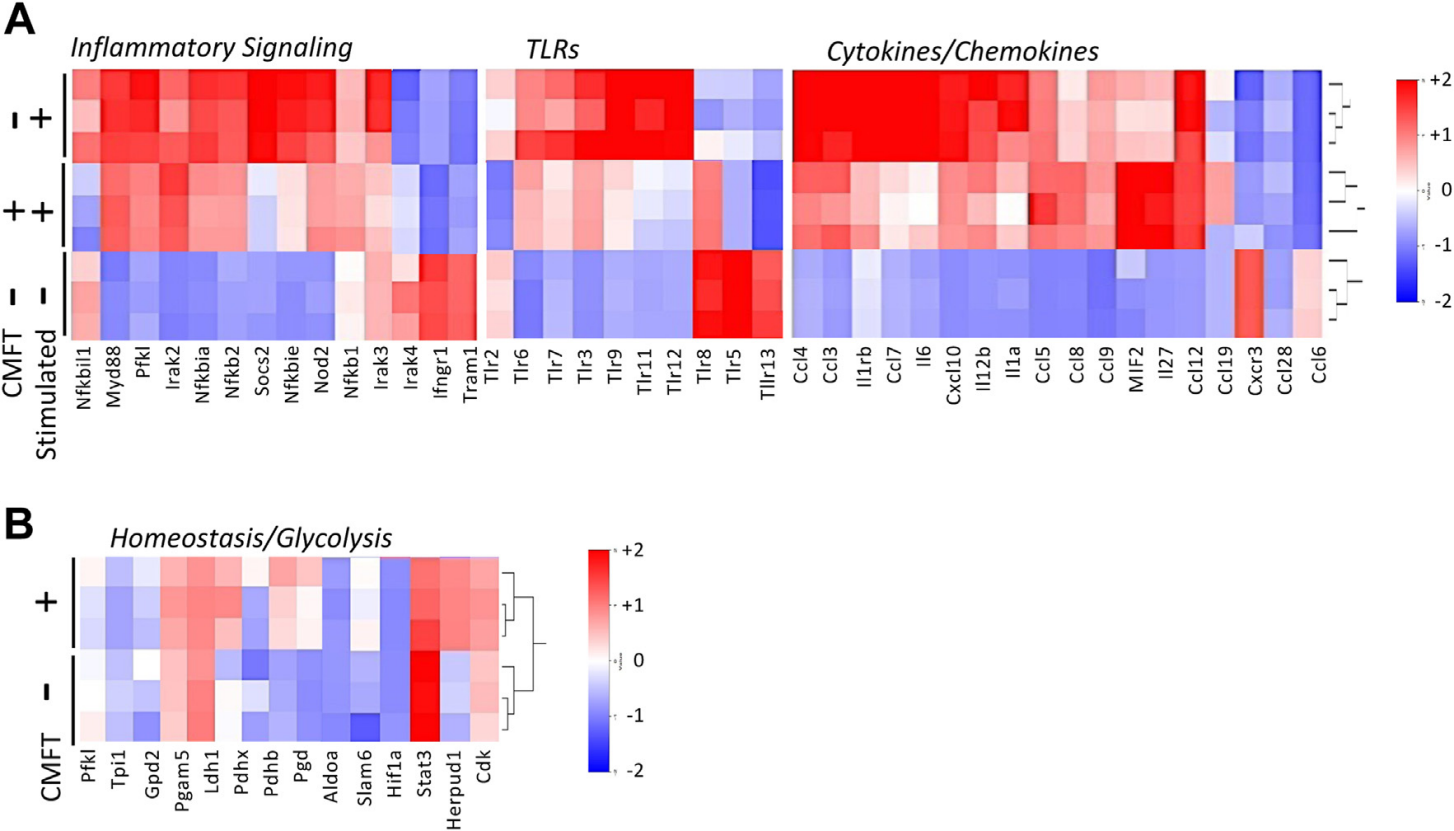
**Transcriptomic analysis of the impact of CMFT on the inflammatory activation of high-genotypic, MIF expressing *MIF***^**CATT7**^**macrophages.** Stimulation of triplicate BMDM cultures were as in Figure 4*B*, with CMFT (+) or vehicle (−) addition for 6 h followed by RNAseq analysis (Agilent Bioanalyzer). Expression heatmaps of responsive genes for 2.0-fold differential expression with an FDR<0.05 in gene expression sets for (*A*): inflammation (Inflammatory Signaling, Toll-like Receptors, and Cytokines/Chemokine) and (*B*): metabolism (Homeostasis/Glycolysis).

Finally, we performed a more comprehensive assessment of gene expression by stimulated *MIF* ^CATT7^ macrophages treated with and without CMFT using RNA-Seq analysis. A bioinformatic analysis employing the MetaCore database was used to identify cellular pathways that were differentially regulated by the addition of CMFT *versus* vehicle control. Immune response-related genes comprised the most significantly downregulated pathways, as expected from previous studies of *Mif* and *ICBP90* genetic knockdown (8). The genes most significantly downregulated by CMFT were representative of three immune pathways: inflammatory signaling, toll-like receptor (TLR) expression, and cytokine/chemokine expression. Expression heatmaps for the genes that were most affected by CMFT are shown in Figure 5*A* together with representative genes within these groupings that were not appreciably regulated by CMFT. Significant downregulation of NFκB (*e.g.*, NFκB p100 and regulatory components), multiple TLRs (*e.g.*, TLR2, TLR3, TLR6, TLR7, TLR9, TLR11, TLR12, TLR13), and cytokines/chemokines (*e.g.*, IL-6, IL-12, IL-1α, IL-1 receptor, CCL6, CCL7, CXCL10) were observed, which agrees with observations in experimental systems of genetic or pharmacologic MIF deficiency (2). We compared these expression results with genes previously reported to be affected by ICBP90 or MIF genetic knockdown in a database of human inflammatory fibroblasts (8). We found *IL1 and IL6* to be significantly reduced by the same fold-expression and FDR criteria in the two independent datasets, confirming prior reports of the MIF-dependence of these two inflammatory cytokines (19, 20). Commensurately, we found CMFT to have no measurable cytotoxicity when assessed by LDH release (% cytotoxicity: *MIF*^CATT5^ BMDMs - LPS: 3.7 ± 0.4%; CMFT: 3.4 ± 0.7%; LPS + CMFT: 1.9 ± 0.3%; *MIF*^CATT7^ BMDMs- LPS: 0 ± 0%; CMFT: 3.0 ± 0.3%; LPS + CMFT: 1.9 ± 0.5%; p = NS for all comparisons). As a more sensitive measure of potential cytotoxicity or dysmetabolic actions, we found no evident impact of CMFT treatment on the expression of a panel of genes involved in metabolic homeostasis conditions (Fig. 4*B*).

## Discussion

Advances in our understanding of the genetic control of immunologic activation pathways prompt consideration of developing agents that may selectively modulate responses based on an individual’s genetic makeup and predisposition to disease (21, 22, 23). In this respect, the functionally polymorphic *MIF* locus, which influences the susceptibility and the severity of autoimmune, infectious, and oncologic diseases, is of interest. Approximately 20% of individuals carry the high-expresser −794 CATT_7_ allele, which confers an increased risk for inflammatory end-organ manifestations by virtue of MIF’s ability to upregulate microbial sensors, sustain downstream inflammatory signaling pathways by inhibiting activation-induced apoptosis and reducing the immunosuppressive action of glucocorticoids (1, 2).

The transcription factor ICBP90 was recently identified to regulate *MIF* expression in a −794 CATT_5-8_ length-dependent manner, with *in vitro* studies showing concordance between the genes influenced by ICBP90 and MIF genetic knockdown, suggesting high specificity of ICBP90 for MIF-dependent responses reliant on inflammatory cytokines, chemokines, and their receptors (8). MIF also is a validated clinical target, with both small molecule and monoclonal antibody-based approaches in advanced clinical testing (24), or in the case of anti-MIF receptor antibody (milatuzumab), clinically approved (25). While MIF antagonism has yet to find an established position in the therapeutic armamentarium, the prevalence of the high-expression −794 *MIF* CATT_7_ allele (*e.g.*, 20%) together with the large effect sizes observed in some diseases or their inflammatory complications (3, 6, 26) offers the opportunity for precision treatment of those individuals who, based on high genotypic *MIF* expression, manifest a MIF-dependent form of disease (3, 4, 6). The availability of such an approach also offers the possibility of streamlining clinical trials, particularly in complex or chronic inflammatory diseases, by pre-selecting genetically susceptible individuals for a more precisely targeted drug with less attendant toxicity. The small-molecule ibudilast for example, while originally developed as a phosphodiesterase inhibitor, has advanced into further clinical testing by virtue of its ability to also inhibit MIF (27). Ibudilast has shown efficacy in multiple sclerosis (24), an autoimmune disease in which a high-expression *MIF* genotype confers risk for progressive disease (5).

As a first step toward the development of a precision or allele-based MIF antagonist, we devised a high-throughput screen aimed at inhibiting the functional interaction between ICBP90 and the *MIF* promoter microsatellite. Using selective target oligonucleotides, an anti-ICBP90 antibody as a positive control, and a sensitive luminescent molecular proximity methodology, we identified CMFT to inhibit ICBP90 binding to the *MIF* promoter microsatellite with an IC_50_ of 470 nM. We further observed CMFT to inhibit *MIF* mRNA expression in two cell-based assays, with evidence for preferential reduction of MIF mRNA and protein in high-genotypic macrophages derived from mice engineered to express the human high- or low-expression *MIF* alleles. Modeling studies of CMFT bound to ICBP90 suggest it has a stronger affinity than the flipped methylated deoxycytidine from the structure of DNA bound to ICBP90 (15). It should be noted that CMFT is in a compound class developed for antibiotic properties but abandoned because of low activity and a poor *in vivo* metabolic profile (28). The present findings nevertheless provide proof of concept for the continued development of CMFT congeners or related molecules to advance the pharmacogenomic development of precision-based MIF inhibitors for diverse autoimmune and inflammatory conditions.

## Experimental procedures

### ICBP90 expression, purification, and activity validation

A recombinant ICBP90 DNA binding (SET and RING finger-associated) domain corresponding to amino acids (aa) 413 to 618 in complex with DNA (15) was cloned into the pET11-b *E. coli* expression plasmid (Novagen), creating a 206 aa C-terminal 6xHis-tagged recombinant protein. The resulting 24.3 kDa protein (ICBP90^413-618^) was purified to homogeneity from *E. coli* lysates (lysis buffer: 20 mM Tris-HCl pH 7.4, 20 mM NaCl, 20 mM imidazole) by fast-protein liquid affinity chromatography using Ni^2+^-nitriloacetic (Ni-NTA) resin and step-wise elution with 20 mM Tris-HCl pH 7.4, 20 mM NaCl, and 500 mM imidazole. Protein purity was verified by SDS-PAGE and western blotting using anti-histidine and anti-ICBP90 antibodies (ab57083, Santa Cruz).

The DNA binding activity of recombinant ICBP90^413-618^ was assessed by melting curve analysis of a double-stranded −794 CATT_8_ *MIF* promoter oligonucleotide (*MIF* gene nucleotides −865 to −752) or control oligonucleotide (−794 CATT_0_ *MIF* promoter DNA, corresponding to nucleotides −833 to −752) (8). The dsDNAs (2.5 pmol) were pre-incubated for 20 min with 10 μl of SYBR Green Dye in a thermocycler (Biorad) and fluorescence emission (λmax = 520 nm at excitation λmax = 497 nm) measured over the temperature range 22°–80 °C after addition of ICBP90^413-618^ or the control proteins: soluble CD74 (sCD74=CD74^73-23^) (17) or human serum albumin (HSA), all tested at 1 to 2 μgs.

High-affinity ICBP90^413-618^- DNA interactions were evaluated by a capture ELISA employing streptavidin high-binding capacity 96-well plates. A 5′biotin-labeled double-stranded −794 CATT_8_ *MIF* promoter oligonucleotide in a concentration range of 0.125 to 2.0 pmol was pre-incubated in 10 μl volumes with 1 nmol ICBP90^413-618^ for 15 min in buffer containing 10 mM Tris-HCl pH 7.5, 50 mM KCl,1 mM DTT. The mixture was added to wells for 60 min, and the plates were blocked with Pierce protein-free T20 blocking buffer (Pierce #37571). After wash steps (TBS, 0.05% Tween-20), the bound complexes were quantified by the addition of anti-ICBP90 and horseradish peroxidase (HRP)-conjugated anti-IgG (PerkinElmer). Competition studies employed unlabeled −794 CATT_8_ *MIF* promoter oligonucleotide.

Electromobility shift assays (EMSA) used the LightShift Chemiluminescent EMSA Kit (ThermoScientific). ICBP90^413-618^ (2 μg) was incubated at 4 °C in 2 μl of 10× binding buffer, 1 μl of 50 ng/μl poly(dI-dC), 11 μl ddH2O, and anti-ICBP90 or control IgG. A 5′ biotin-labeled *MIF* promoter CATT_8_ oligonucleotide (20 fmol) was added to the reaction mixture with or without unlabeled excess *MIF* promoter CATT_8_ or CATT_0_ oligonucleotides (4 pmol) and incubated for 20 min at 22 °C. Samples were electrophoresed at room temperature using 6% (w/v) non-denaturing polyacrylamide gels prior to transfer onto nylon membranes for chemiluminescence detection. Test compounds were screened at concentrations of 5 to 40 μM.

### High-throughput screening

An amplified luminescent proximity homogeneous assay (AlphaScreen Histidine Detection Kit #6760619C, PerkinElmer) (12) was employed using a PerkinElmer Envision plate reader (29, 30). We evaluated assay performance by varying buffer conditions under multiple concentrations of the *MIF* promoter CATT_8_ oligonucleotide and ICBP90^413-618^ protein (6.8–5000 nM each). The selected assay buffer was 10 mM Tris pH 7.5, 50 mM KCl, 1 mM DTT, 0.01% Tween20, 0.1% BSA with 10 nM oligonucleotide and 21 nM ICBP90^413-618^ as optimal. The storage buffer for oligonucleotide was 10 mM Tris pH 8, 1 mM EDTA, and for ICBP90^413-618^, 10 mM Tris pH 8, 0.5 M NaCl, 5% glycerol, and 2 mM DTT. Compound screening was in 10 μl reaction volumes comprising 20 nM ICBP90^413-618^ (21 nM, 4 μl) pre-incubated for 15 min with test compounds (40, 20, 10, 5 μM in 20 nl) followed by the addition of *MIF* promoter CATT_8_ oligonucleotide (10 nM, 2 μl) with incubation for 15 min, and the addition of Alpha Screen beads (4 μl) for 60 min. The positive plate control was an anti-ICBP90 antibody (25 nM) and the negative control was a compound solvent (0.4% DMSO). Raw data was normalized to percent effect with the positive control set as 100% inhibition of ICBP90^413-618^ - *MIF* promoter oligonucleotide CATT_8_ binding. Positively scoring compounds were further tested for interference with the AlphaScreen fluorescence signal (*e.g.*, biotin mimetics) using the AphaScreen PerkinElmer TrueHitsKit (#6760627D).

For pilot screening and assay validation, the Microsource Pharmakin library (1600 compounds) was used, producing a Z’ = 0.84 to 0.93. An activity cutoff of median signal ± 3 SDs of compounds at 40 μM yielded a positive rate of ∼1.7%. A total of 29,000 compounds were screened from the ChemBridge, ChemDIV, Enzo, Microsource, NCI, and NIH clinical source small molecule collections at the Yale Center for Molecular Discovery https://ycmd.yale.edu/smallmoleculecollections.

### Structural modeling

Computational modeling employed the SRA domain (structure 3CLZ.pdb, 2.2 Å) of ICBP90 (15) and AutoDock, a program for docking ligands to receptors and for predicting binding mode and affinity (31). Protein preparation for computational studies involved DNA removal from the binding site, addition of protein hydrogens, bond optimization, and energy minimization. Subsequently, a docking grid box was created for the docking process. The docking grid box was centered at the Trp32 residue with a box size of 36.73 × 19.46 × 32.97 Å that covers the entire binding pocket of the methylated DNA with a grid spacing of 0.375 Å. The ligand CMFT also was prepared using AutoDock utilities. The ligand docking was performed against the SRA domain using AutoDock4 (31). The obtained poses were analyzed using cutoff docking scores and visually inspected for a precise understanding of intermolecular interactions between the protein and ligand. All poses were visualized using PyMol (www.pymol.org). The hydrogen bond interactions in a compound-protein binding were analyzed using the PLIP online engine (https://doi.org/10.1093/nar/gkab294).

### Cell-based activity assays

*MIF* -794 CATT_5-8_ dependent mRNA transcription was first assessed using four corresponding *MIF* promoter/luciferase reporter plasmids and an isologous *MIF* -794 CATT_0_ plasmid control as described previously (8). One μg of each *MIF* reporter plasmid together with a β−actin Renilla luciferase plasmid was used per transfection of cultured human THP-1 monocyte cells utilizing the lipofectamine 2000 reagent (Invitrogen). Transfected monocytes were stimulated with lipopolysaccharide (LPS, 100 ng/ml; *E. coli* serotype 0111:B4, Sigma-Aldrich) and simultaneously treated with test compounds or vehicle control (0.4% DMSO), and the luciferase activity assessed 6 h later by Dual-Luciferase assay (Promega). Transfected THP-1 cell line responses were monitored for uniformity over time and verified by human TNF release (8).

The development and validation of two humanized *MIF* mouse strains created by recombinant replacement of mouse *Mif* with the human low (−794 CATT_5_) and high (−794 CATT_7_) expression *MIF* alleles have been described previously: [C57BL/6NTac-Miftm3883.1(MIF)Tac-Tg(CAG-Flpe)2Arte] and [C57BL/6NTac-Miftm3884.1(MIF)Tac-Tg(CAG-Flpe)2Arte] mice (6, 32). Bone marrow-derived macrophages (BMDMs) were isolated from these *MIF* ^CATT5^ and *MIF* ^CATT7^ mice and 1 x 10^6^ cells per well cultured in DMEM, 10% FBS prior to stimulation with LPS (100 ng/ml, 6 h) and treatment with CMFT (2.5 μM) or vehicle (0.4% DMSO). Cells were collected, lysed, the RNA was extracted using the RNeasy extraction kit (Qiagen), and cDNA was synthesized from 1 μg RNA (iScript cDNA Synthesis Kit, Bio-Rad). Real-time PCR was carried out with the iQ SYBR Green system (Bio-Rad) and nucleotide primers for MIF (33). The emitted fluorescence for each reaction was measured during the annealing/extension phase and relative quantity values were calculated by the standard curve method. The quantity value of GAPDH in each sample was used as a normalizing control. Data were analyzed with the comparative cycle time (CT) method. MIF protein release into BMDM culture supernatants was measured by specific ELISA (8). Cellular cytotoxicity was measured by supernatant lactate dehydrogenase content (LDH-Cytox Assay, Biolegend).

RNA-Seq and differential gene expression analysis were performed individually on stimulated and treated BMDMs from triplicate samples (34). Total RNA was extracted using the RNeasy Plus Mini MinElute Cleanup Kit (Qiagen) and the RNA quality was determined by estimating the A_260/_A_280_ and A_260_/A_230_ ratios using a NanoDrop spectrophotometer (Thermo Fisher Scientific). The RNA integrity was verified by Agilent 2100 Bioanalyzer based on the relative abundance of 18S and 28S rRNA. Eighteen sequencing libraries were produced by the Illumina TruSeq stranded protocol for 76-bp paired-end sequencing using Illumina HiSeq 2500. Adapter sequences, empty reads, and low-quality sequences were removed. The nucleotides at the 5′ and 3′ end with a quality score below 20 for each read were trimmed using in-house scripts and read pairs with either end shorter than 45 bp after trimming were discarded. Reads passing quality control were aligned using Tophat v.2.0.13 (53) to perform spliced alignment of the reads against the reference UCSC mouse genome and transcript annotation. Only the reads that mapped to a single unique location within the genome and with a maximum of 2 mismatches in the anchor region of the spliced alignment were reported in these results. We used the default settings for all other Tophat options. Tophat alignments then were processed by Cufflinks v2.2.1 1 (35) to quantify the abundance of each transcript. The transcript abundance was measured in fragments per kb of exon per million mapped fragments (FPKM) to normalize the read count of a transcript by both its length and library size. These normalized transcript abundances then were analyzed to identify differential gene expression between conditions using Cuffdiff (36) with default options. After differential gene expression analysis, the significantly differentially expressed genes in the LPS-stimulated, CMFT-treated samples were chosen using a cutoff of FDR-controlled *p* value less than 0.05. The significantly different genes identified from LPS-stimulated *versus* unstimulated groups were compared with those identified from CMFT-treated *versus* untreated groups, and overlapping genes were analyzed for cellular pathways within the MetaCore database related to inflammatory signaling, Toll-like receptor activation, and cytokine/chemokine expression. FPKM values of differentially expressed genes were visualized in heatmaps and the z score was normalized using the R package. The gene expression data files are available upon publication in the International MIF Consortium database (http://www.biochemmcb.rwth-aachen).

### Statistics

Data are representative of at least 3 independent experiments (unless stated otherwise), and statistical analyses were conducted using GraphPad Prism software. Results are expressed as mean ± SD. Statistical tests for each graph are described in figure legends and *p* values of less than 0.05 were considered significant.

### Study approval

All experimental procedures involving experimental mice were approved by the Yale University IACUC and conducted in accordance with the IACUC and AAALAC guidelines.

## Data availability

The gene expression data files are available upon publication in the International MIF Consortium database (http://www.biochemmcb.rwth-aachen).

## Conflict of interests

The authors declare that they have no conflicts of interest with the contents of this article.
